# American Medical Association

**Published:** 1882-06-20

**Authors:** 


### *AMERICAN MEDICAL ASSOCIATION.*

As we go to press we have not received the full proceedings of the American Medical Association, which assembled, the present year, at St. Paul, Minnesota. As was anticipated, the late action of the New York Medical Society in relaxing the code of ethics in the matter of consultations was denounced,and the old established code reaffirmed. The right of the New York deligates to seats in the Association was early introduced and referred to the Judicial Council, who reported adversely amid loud and repeated applause of the body.

Several State societies have taken similar action, condemning the action of the New York society, and nearly all the medical journals have likewise denounced the measure. The New York Medical Record being almost alone in its support of the new departure.

